# Semi-quantitative MALDI-TOF for antimicrobial susceptibility testing in *Staphylococcus aureus*

**DOI:** 10.1371/journal.pone.0183899

**Published:** 2017-08-31

**Authors:** Tucker Maxson, Cheryl L. Taylor-Howell, Timothy D. Minogue

**Affiliations:** Diagnostic Systems Division, United States Army Medical Research Institute of Infectious Disease, Fort Detrick, Maryland, United States of America; Universitatsklinikum Munster, GERMANY

## Abstract

Antibiotic resistant bacterial infections are a significant problem in the healthcare setting, in many cases requiring the rapid administration of appropriate and effective antibiotic therapy. Diagnostic assays capable of quickly and accurately determining the pathogen resistance profile are therefore crucial to initiate or modify care. Matrix-assisted laser desorption/ionization time-of-flight (MALDI-TOF) mass spectrometry (MS) is a standard method for species identification in many clinical microbiology laboratories and is well positioned to be applied towards antimicrobial susceptibility testing. One recently reported approach utilizes semi-quantitative MALDI-TOF MS for growth rate analysis to provide a resistance profile independent of resistance mechanism. This method was previously successfully applied to Gram-negative pathogens and mycobacteria; here, we evaluated this method with the Gram-positive pathogen *Staphylococcus aureus*. Specifically, we used 35 strains of *S*. *aureus* and four antibiotics to optimize and test the assay, resulting in an overall accuracy rate of 95%. Application of the optimized assay also successfully determined susceptibility from mock blood cultures, allowing both species identification and resistance determination for all four antibiotics within 3 hours of blood culture positivity.

## Introduction

Antimicrobials are effective and often life-saving treatment options for bacterial infections; however, rising levels of resistance and the emergence of multidrug-resistant strains are becoming major challenges in continuing to combat these pathogens. Researchers and medical professionals are pursuing several avenues for countering these problems, with increased antimicrobial stewardship among the forefront [1]. Utilizing antimicrobials in a more conservative fashion and de-escalating initial broad-spectrum treatment when appropriate will help maintain the effectiveness of the current antimicrobial arsenal [2, 3]. However, the severe deterioration of clinical outcomes associated with delayed or ineffective antimicrobial treatment, especially in cases of septicemia, dictate that an effective treatment regimen be initiated as quickly as possible [4]. Therefore, diagnostic platforms that provide both rapid pathogen identification and antimicrobial susceptibility testing (AST) are crucial to providing adequate patient care and for the implementation of stewardship programs.

Matrix-assisted laser desorption/ionization time-of-flight (MALDI-TOF) mass spectrometry (MS) is an effective tool for routine bacterial and yeast identification, with increasing adoption in clinical microbiology laboratories due to its fast time to answer and low per-sample costs [5, 6]. The application of MALDI-TOF MS for AST would provide further utility for this technique and allow rapid identification and susceptibility testing of pathogens with a single assay. Several ongoing efforts investigate methods for determining antimicrobial resistance via MALDI-TOF MS [7, 8], including monitoring antibiotic modification by bacterial culture (e.g., β-lactam hydrolysis [9, 10], acetylation of fluoroquinolones [11]), direct detection of proteins involved in specific resistance mechanisms [12, 13], and detection of stable isotope labeling [14, 15]. However, the former two methods rely on identification of a specific resistance mechanism and are only applicable with certain antibiotic classes, while the latter case requires expensive, isotopically labeled media.

Recent studies reported an alternative MALDI-TOF MS based method for AST termed the MALDI Biotyper antibiotic susceptibility test rapid assay (MBT-ASTRA) as a possible solution to these limitations [16, 17]. MBT-ASTRA utilizes semi-quantitative MALDI-TOF MS to measure the relative growth rates of bacterial isolates exposed to antibiotic compared to untreated controls during a short incubation step. Samples are spiked with a reference standard to allow normalization across spectra, and the total area under the curve (AUC) of all peaks within a defined mass range is calculated to provide a measure of bacterial growth. In this method, a significantly reduced AUC generated from antibiotic treated samples relative to those from untreated controls indicates susceptibility to the antibiotic. Since the MBT-ASTRA assay utilizes growth rate information for resistance determination, it benefits from functioning independently of the mechanism of resistance and potentially can be utilized with any antibiotic. The assay also does not require specialized media or instrumentation, beyond the MALDI-TOF mass spectrometer.

One drawback of the MBT-ASTRA assay is the concentration of antibiotic used and the incubation time must be optimized for each species and antibiotic combination [18], hindering its easy adoption as a generalized diagnostic tool. Thus far, the assay has been applied to a range of clinically relevant Gram-negative species and to mycobacteria [18, 19], but notably absent is data demonstrating efficacy with any Gram-positive species. Given the clinical importance of many Gram-positive pathogens and the known difficulties associated with their sample preparation for MALDI-TOF analysis [5, 20], it is imperative to determine if MBT-ASTRA can be extended to Gram-positive species as well. The goal of this study is to bridge this gap utilizing *Staphylococcus aureus*, one of the Gram-positive members of the so-called ESKAPE pathogens (*E**nterococcus faecium*, *S*. *aureus*, *K**lebsiella pneumoniae*, *A**cinetobacter baumannii*, *P**seudomonas aeruginosa*, and *E**nterobacter* species). The ESKAPE pathogens are a group of bacteria identified as clinically important due to high resistance rates and their prevalence in nocosomial infections. Here, we describe the optimization and functionality of a Gram-positive assay for four antibiotics with optimized assay conditions successfully applied to determine resistance profiles directly from spiked blood culture.

## Materials and methods

### Bacterial isolates and culture

Bacterial isolates were obtained from the American Type Culture Collection (ATCC), the Biological and Emerging Infections Resources Program (BEI Resources), the Children’s National Medical Center via the Food and Drug Administration (FDA), and the Centers for Disease Control (CDC). The origins of each strain are listed in Table 1. Bacteria were propagated on Mueller-Hinton (MH) agar plates and cultured in tryptic soy broth (TSB) liquid medium at 37°C unless otherwise noted. Antibiotics were obtained from Sigma-Aldrich or Gold Biotechnology and used at the concentrations listed from 100x stock solutions in water.

**Table 1:** Isolate sources and MICs.

*S*. *aureus* subsp.	Source	Ciprofloxacin MIC (ug/mL)	Oxacillin MIC (ug/mL)	Cefepime MIC (ug/mL)	Vancomycin MIC (ug/mL)
BAA-38	ATCC	0.19	16	64	2
BAA-39	ATCC	4	48	24	2
BAA-40	ATCC	0.125	>256	192	2
BAA-41	ATCC	12	>256	>256	2
BAA-43	ATCC	16	>256	>256	2
BAA-44	ATCC	12	>256	>256	2
700698	ATCC	>32	>256	>256	3
FDAARGOS_135	CNH	8	128	192	2
FDAARGOS_140	CNH	6	128	192	2
GRIM 48	CNH	0.19	0.5	3	1.5
FDAARGOS_159	CNH	0.19	0.5	2	2
FDAARGOS_169	CNH	1	32	64	1.5
ATCC 25923	ATCC	0.19	0.19	1.5	1.5
ATCC 29213	ATCC	0.19	0.25	2	1.5
ATCC 12600	ATCC	0.19	0.19	2	1
ATCC 13565	ATCC	0.19	0.25	1.5	0.75
ATCC 14458	ATCC	0.125	1	2	1.5
ATCC 8095	ATCC	0.19	0.19	2	1
ATCC 8096	ATCC	0.125	0.125	1.5	1.5
ATCC 29247	ATCC	0.125	0.38	2	1.5
HIP13170	BEI	>32	>256	>256	4
HIP13419	BEI	>32	256	>256	4
880 (BR-VRSA)	BEI	>32	128	128	>256
AIS 1000505	BEI	>32	>256	>256	6
71080	BEI	>32	>256	>256	>256
VCU089	BEI	32	0.125	2	1.5
HFH-29568	BEI	8	16	24	1.5
MN8	BEI	0.25	0.38	3	1.5
HT 20020058	BEI	0.25	0.75	2	1.5
HT 20020470	BEI	0.25	0.38	2	1.5
A970675	BEI	0.19	0.75	3	1.5
MNHOCH	BEI	0.25	0.75	3	1.5
VCU006	BEI	0.19	0.19	2	2
HFH-30123	BEI	>32	>256	>256	2
A890259	BEI	0.19	0.19	3	1.5
AR# 0215	CDC				4
AR# 0216	CDC				4
AR# 0217	CDC				8
AR# 0218	CDC				4
AR# 0219	CDC				8
AR# 0220	CDC				8
AR# 0221	CDC				4
AR# 0222	CDC				4
AR# 0223	CDC				4
AR# 0224	CDC				4
AR# 0225	CDC				4
AR# 0226	CDC				4
AR# 0227	CDC				4
AR# 0228	CDC				4

ATCC, American Type Culture Collection; CNH, Children’s National Hospital; BEI, Biodefense and Emerging Infections Research Resources Repository; CDC, Centers for Disease Control

### Standard resistance determination

Minimum inhibitory concentrations (MICs) were determined by Etest. Freshly plated bacteria were grown for approximately 24 h at 37°C before colonies were used to create 0.5 McFarland suspensions in saline solution. The solutions were used to uniformly inoculate MH agar plates via polyester swabs. Etest strips (bioMérieux, France) were placed on the plates and incubated for 24 h at 37°C. MIC values were determined from the test strips as per the manufacturer’s instructions.

### Sample preparation for quantitative MALDI-TOF

Cultures of *S*. *aureus* prepared in TSB liquid media were inoculated from colonies on fresh MH plates and incubated at 37°C with shaking overnight (~16 h). The cultures were diluted 1:100 with fresh TSB and incubated an additional 2 h before being further diluted to a density of McFarland 0.5. For each replicate for each strain, two 200 μL aliquots in 1.7 μL Eppendorf tubes were prepared with one containing no antibiotic and the other containing the optimized concentration of antibiotic (4 μg/mL for ciprofloxacin, 2 μg/mL for oxacillin, 4 μg/mL for cefepime, or 2 μg/mL for vancomycin). The samples were incubated for 2 h at 37°C with shaking in a ThermoMixer (Eppendorf, Germany). After incubation, the samples were centrifuged at 16,000 x g at room temperature for 3 min and the supernatant was removed. The pellets were washed once with 1 mL of an aqueous solution of 70% ethanol, with special care being given to remove all residual ethanol. The tubes were left open to dry for an additional 3–5 minutes before 40 μL of 70% formic acid was used to resuspend the bacterial pellets. Subsequently, 40 μL of acetonitrile containing 0.2 mg/mL RNase A (as a reference standard) was added and the samples were briefly mixed by vortex before being centrifuged at 16,000 x g for 3 min at room temperature. Samples were either used for MALDI-TOF analysis immediately or were stored at -80°C for up to 5 days without any significant degradation in quality.

### MALDI-TOF analysis

One microliter of formic acid protein extract was spotted on a polished steel MALDI target and allowed to completely dry. One microliter of a 5 mg/mL solution of α-cyano-4-hydroxy-cinnamic acid (CHCA) in 50% acetonitrile—47.5% water—2.5% trifluoroacetic acid was then spotted over the dried spots within 30 min. After drying, the samples were analyzed with either a Microflex LRF or an Autoflex III smartbeam MALDI-TOF MS instrument (Bruker Daltonics, Bremen, Germany) in linear positive mode. Both instruments produced equally usable data. Instrument parameters for the Microflex were: mass range, 2,000–20,800 Da; ion source (IS) 1, 20.0 kV; IS2, 18.35 kV; lens, 9.0 kV; detector gain, 2,916 V; deflection up to 1,950 Da. Instrument parameters for the Autoflex were: mass range, 2,000–20,800 Da; ion source (IS) 1, 20.0 kV; IS2, 18.35 kV; lens, 7.0 kV; detector gain, 1868 V; gating, 2,000 Da; gating strength, high. Spectra were acquired at maximum frequency with 2,000 shots per spot. Instrument calibration was performed with the bacterial test standard (BTS; Bruker Daltonics). All samples were analyzed in duplicate.

### Data evaluation

**Fig 1:**
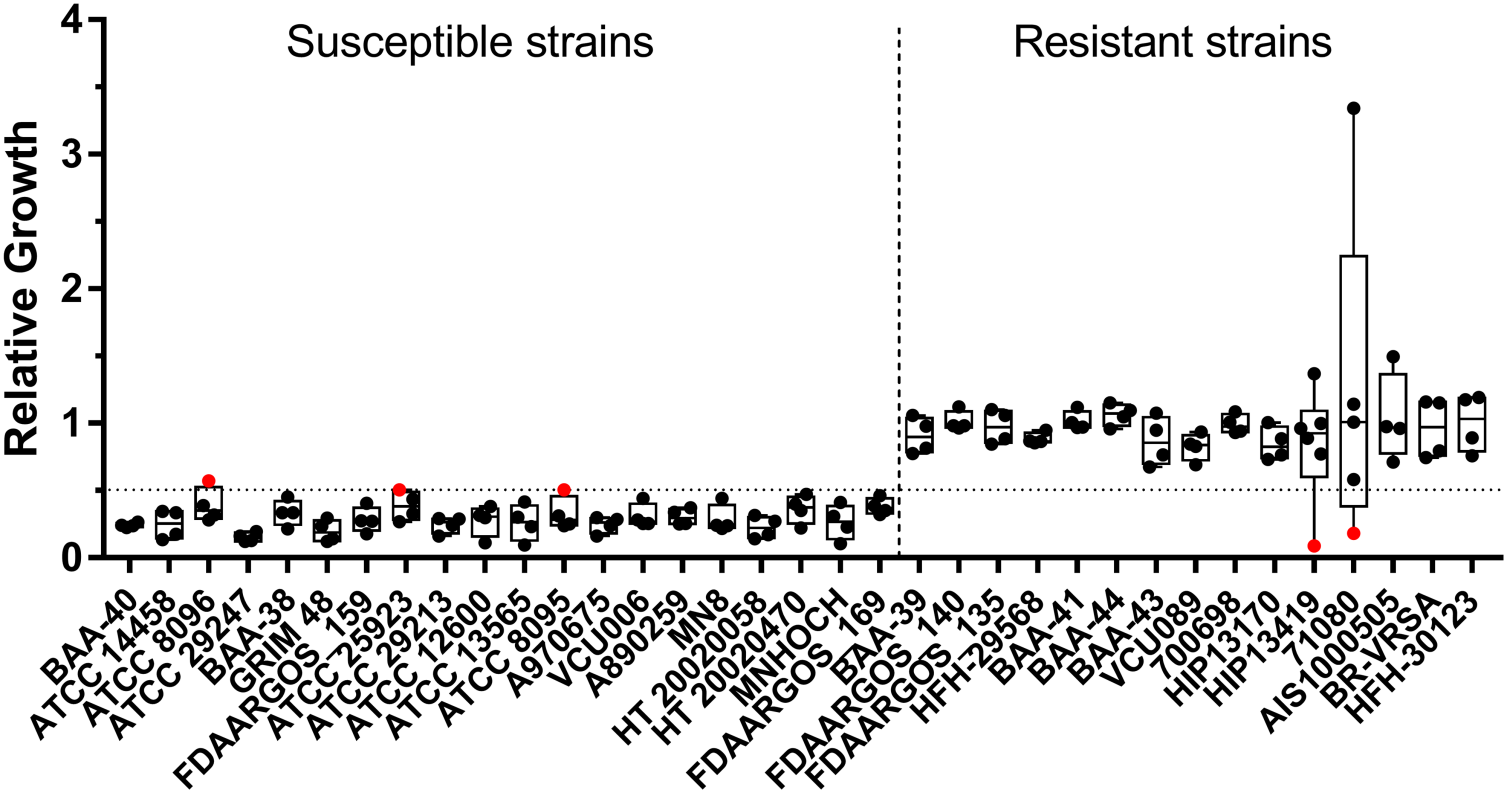
Assay performance with ciprofloxacin. Relative growth values for 35 *S*. *aureus* strains derived from the MBT-ASTRA assay performed with ciprofloxacin are shown. A relative growth cutoff of 0.5 was utilized to classify resistance, indicated by the horizontal line. Strains are arranged from left to right in order of increasing MIC, with exact values given in Table 1. Data points colored red indicate major or very major errors. For each strain, 4–6 independent replicates were obtained on different days.

**Fig 2:**
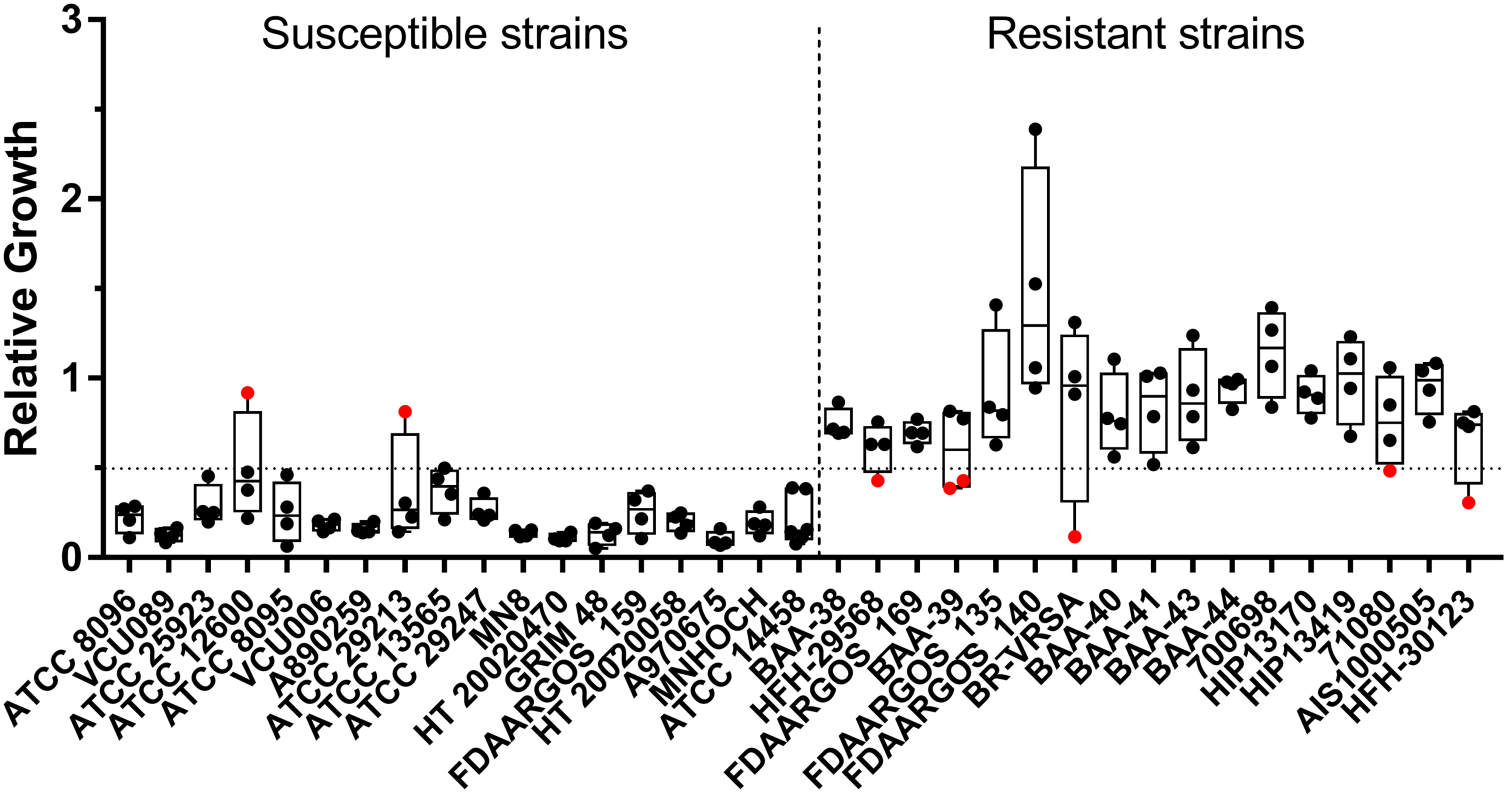
Assay performance with oxacillin. Relative growth values from the MBT-ASTRA assay performed with oxacillin are shown. A relative growth cutoff of 0.5 was utilized to classify resistance, indicated by the horizontal line. Strains are arranged from left to right in order of increasing MIC, with exact values given in Table 1. Data points colored red indicate major or very major errors. For each strain, 4–6 independent replicates were obtained on different days.

**Fig 3:**
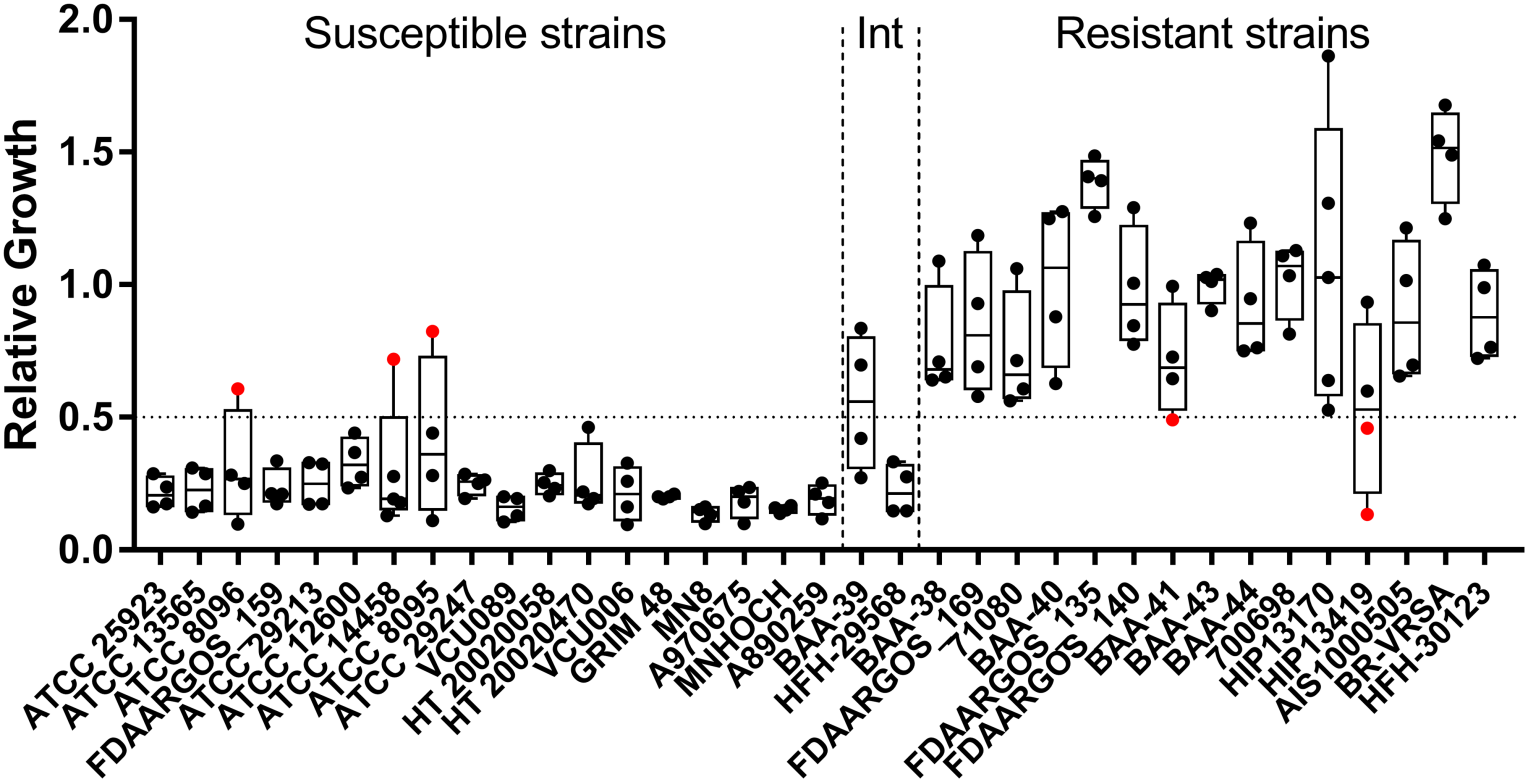
Assay performance with cefepime. Relative growth values from the MBT-ASTRA assay performed with cefepime are shown. A relative growth cutoff of 0.5 was utilized to classify resistance, indicated by the horizontal line. Intermediate resistance is abbreviated as Int. Strains are arranged from left to right in order of increasing MIC, with exact values given in Table 1. Data points colored red indicate major or very major errors. For each strain, 4–5 independent replicates were obtained on different days.

**Fig 4:**
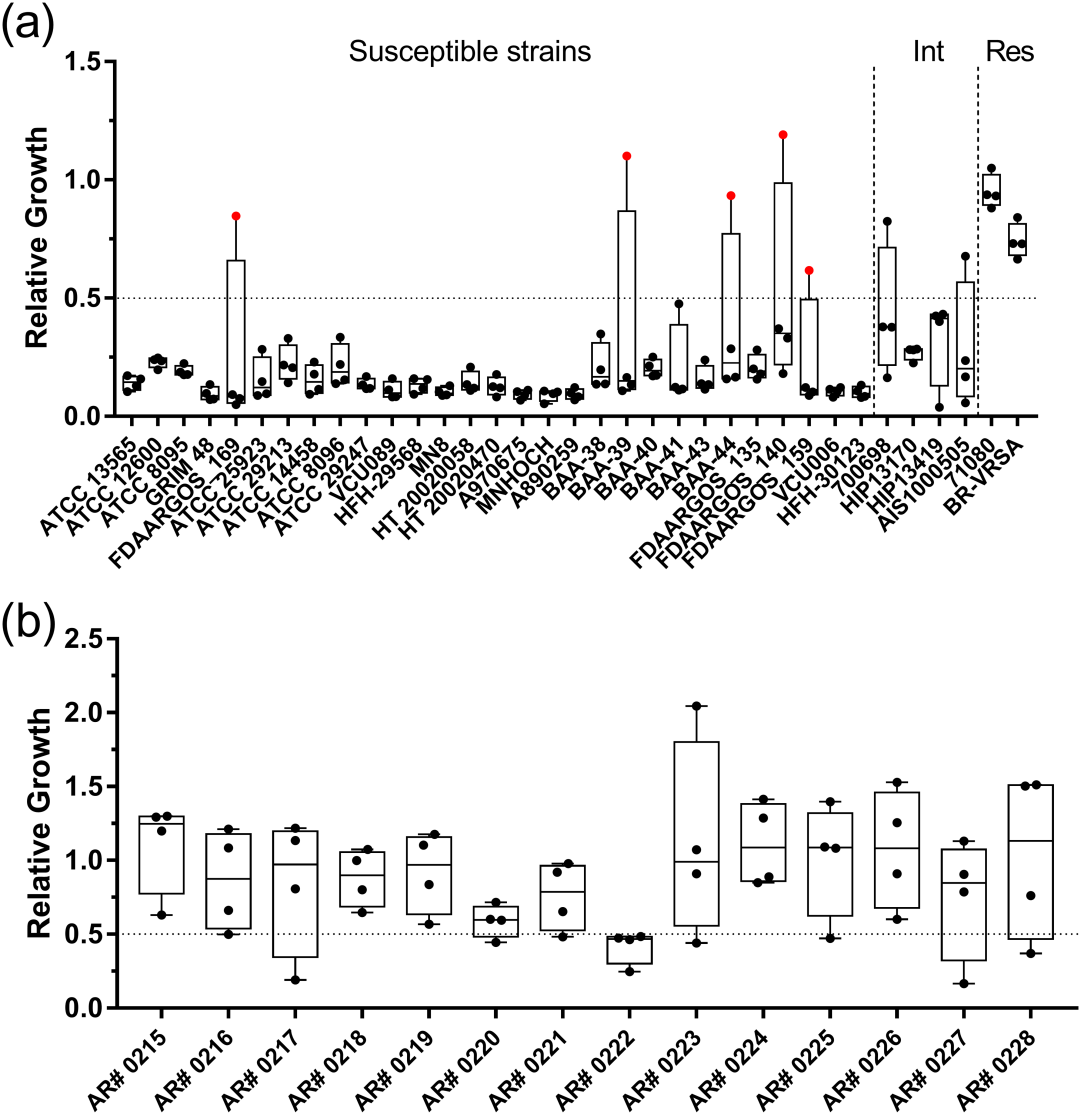
Assay performance with vancomycin. Relative growth values from the MBT-ASTRA assay performed with vancomycin for the 35 *S*. *aureus* strains in the original panel (a) and an additional 14 VISA isolates (b) are shown. A relative growth cutoff of 0.5 was utilized to classify resistance, indicated by the horizontal line. Intermediate resistance is abbreviated as Int and full resistance as Res. Strains are arranged from left to right in order of increasing MIC in panel (a), with exact values given in Table 1. Data points colored red indicate major errors. For each strain, 4 independent replicates were obtained on different days.

**Fig 5:**
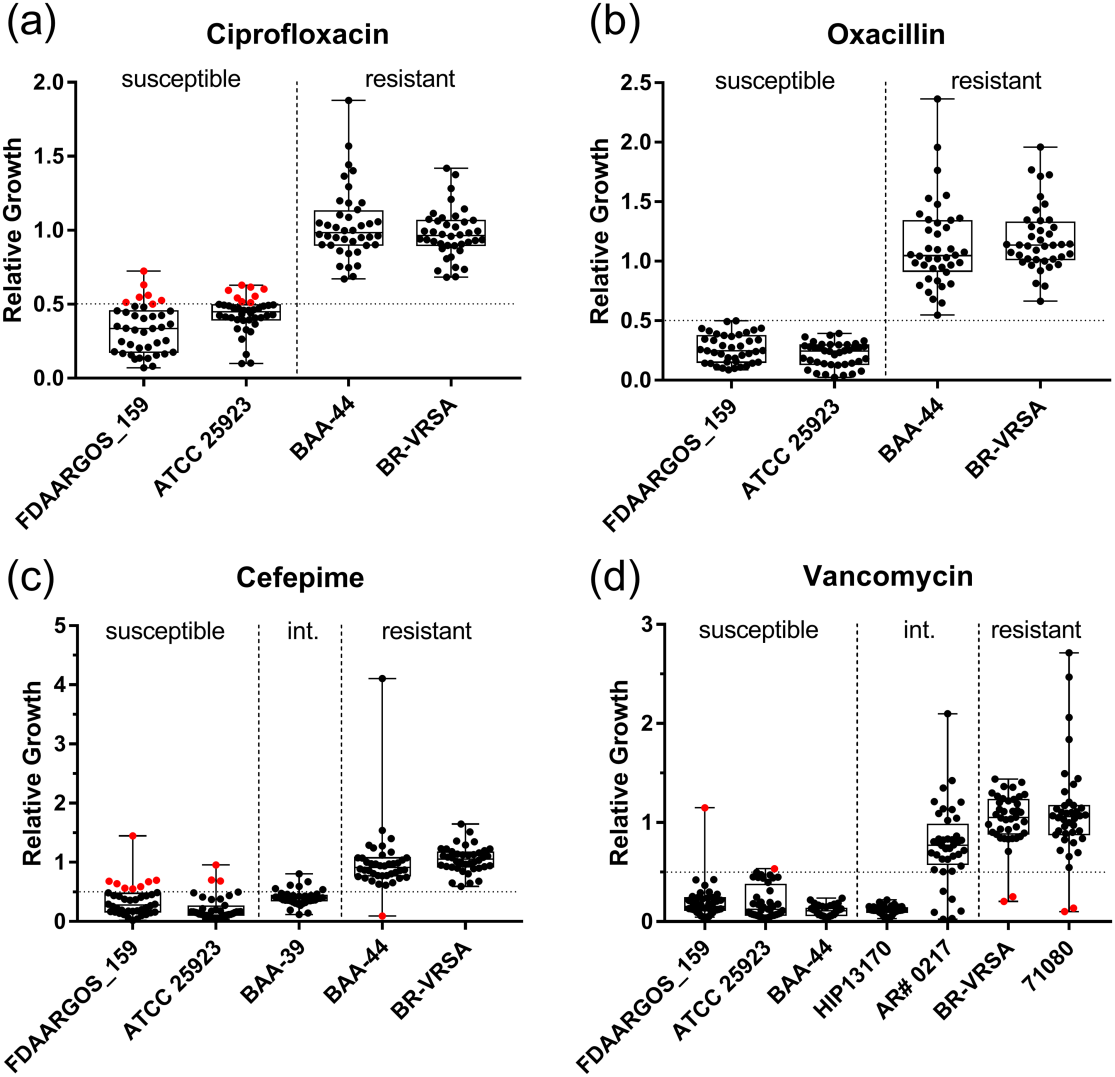
Assay reproducibility. Assay reproducibility from 40 replicates with strains susceptible and resistant to each antibiotic. Relative growth values from each replicate are shown, with data points representing major and very major errors colored red. A relative growth cutoff of 0.5 was utilized to classify resistance, indicated by the horizontal line. Intermediate resistance is abbreviated as Int. For each strain, 40 replicates were obtained over at least 4 days with up to 12 replicates from a single starter culture.

Automated data analysis was performed with the MBT-ASTRA software prototype made available by Bruker [16, 19]. Briefly, the software normalizes the peaks, performs peak picking, and determines the AUC of each spectrum. A mass range of 4,000 to 10,000 Da was used in the analysis. Relative growth values are calculated as the ratio of AUC from the antibiotic treated sample to the AUC from the no antibiotic control. The analysis is performed with each combination of the duplicate spots analyzed for each sample, resulting in four values that are then averaged to give the relative growth for the replicate. A cutoff value of 0.5 was used to distinguish between susceptible and resistant isolates. Resistance breakpoints were based on current guidelines from the Clinical & Laboratory Standards Institute (CLSI) [21], except for cefepime which was based on an older version of the guidelines as it has been removed from the current document [22]. Data is displayed with box and whisker plots in Figs 1–5; the medians are indicated by the center lines, the minima and maxima are indicated by whiskers, and the 25^th^ and 75^th^ percentiles are indicated by boxes. Sensitivity was defined as the number of true positives (resistant by assay and reference standard) over total positives (resistant by reference standard). Specificity was defined as the number of true negatives (susceptible by assay and reference standard) over total negatives (susceptible by reference standard). Accuracy was defined as number of true positives and true negatives over the total number of replicates (total positive plus total negative). Binomial proportion confidence intervals were calculated with the Clopper-Pearson interval using symmetrical tails.

### Spiked blood culture

All blood cultures were performed with BD BACTEC standard/10 aerobic/F bottles and were incubated in a BD BACTEC FX40 instrument. Blood cultures were inoculated with 10 mL of fresh human whole blood (BioreclamationIVT, Maryland, USA) spiked with 400 μL of *S*. *aureus* culture in TSB. The initial bacterial concentrations at inoculation were approximately 10^6^ CFU/mL and bacterial densities upon positivity were in the range of 10^7^ to 10^8^ CFU/mL, which is in line with previously reported densities from blood culture bottles inoculated with clinical samples [23]. The high initial inoculum was utilized to allow experiments to be completed within one day. Bottles were incubated until they flagged positive (~3.5 h) and 500 μL of culture was removed within 30 minutes of positivity. The culture was diluted 1:4 with fresh TSB, and then used to make five 200 μL aliquots. For each aliquot, either 4 μg/mL ciprofloxacin, 2 μg/mL oxacillin, 4 μg/mL cefepime, 2 μg/mL vancomycin, or no antibiotic was added and the samples were incubated at 37°C with shaking in a ThermoMixer for 2 h. After incubation, 40 μL of 10% SDS in water was added to each sample and the samples were vortexed for 10 s to lyse blood cells. The samples were then centrifuged at 16,000 x g for 3 minutes at room temperature and the supernatant was carefully removed. The pellets were washed with 1 mL of water, and were then processed the same as above, starting at the 70% ethanol wash step.

## Results

Previous work demonstrated that the antibiotic concentration and incubation time used in the MBT-ASTRA assay must be optimized for each antibiotic / species combination, with the discriminating antibiotic concentration potentially being significantly different than the resistance breakpoint concentration [18]. The following three subsections show results obtained from applying the optimized conditions to 35 strains of *S*. *aureus* (≥ 4 independent replicates on different days) for four antibiotics. Assay accuracy assessment for each case, based on comparison to Etest results (Table 1) as a reference standard, showed sensitivities, specificities, and overall accuracies ranging from 91–100% (Table 2).

**Table 2:** Summary of MBT-ASTRA assay performance across 35 strains.

Antibiotic	Isolates resistant by Etest		Isolates susceptible by Etest		Total replicates	Very major error rate	Major error rate	Sensitivity (95% CI)^a^	Specificity (95% CI)^a^	Overall accuracy
	Susceptible by MBT-ASTRA	Resistant by MBT-ASTRA	Susceptible by MBT-ASTRA	Resistant by MBT-ASTRA						
Cirpofloxacin	2	61	77	3	143	1.4%	2.1%	97% (89–100)	96% (89–99)	97%
Oxacillin	6	62	72	2	142	4.2%	1.4%	91% (82–97)	97% (91–100)	94%
Cefepime	3	58	70	3	134	2.2%	2.2%	95% (86–99)	96% (89–99)	96%
Vancomycin	0	8	111	5	124	0.0%	4.0%	100% (63–100)	96% (90–99)	96%
Overall	11	189	330	13	543	2.0%	2.4%	95% (90–97)	96% (94–98)	96%

^a^CI, confidence interval

### Ciprofloxacin resistance detection

For ciprofloxacin, a concentration of 4 μg/mL was the lowest concentration providing consistent differentiation between resistant and susceptible strains, while an incubation time of 2 h was required to allow sufficient growth of slower growing *S*. *aureus* strains. Incubation times longer than 2 h resulted in an overgrowth of the faster growing strains, often leading to what appeared to be poor protein extraction during the formic acid workup step. This manifested as a visibly large cell mass that, when subjected to extraction and analysis by MALDI-TOF MS, produced little to no signal from cell-derived protein while the spiked-in reference standard appeared with high intensity. Extraction failure still occasionally occurred with a 2 h incubation but at a lower rate, likely accounting for a portion of the errors recorded in this study. Applying the optimized assay conditions to all 35 strains, an overall accuracy of 97% (138/143 total replicates) was obtained, with 3 major errors (defined as reference result is susceptible and assay result is resistant) and 2 very major errors (reference result is resistant and assay result is susceptible) (Table 2, Fig 1, S1 Fig). This included the correct classification of both a susceptible (FDAARGOS_169, MIC = 1 μg/mL) and a resistant (BAA-39, MIC = 4 μg/mL) isolate with MICs falling just below and above the breakpoint concentration (CLSI guidelines, ≤ 1 μg/mL classified as susceptible, ≥ 4 μg/mL classified as resistant).

### β-lactam resistance detection

We chose oxacillin and cefepime as representatives of the penicillin and cephalosporin families of β-lactam antibiotics, respectively. As with ciprofloxacin, 2 h incubation yielded the best assay performance for the reasons presented above. For oxacillin, an antibiotic concentration of 2 μg/mL was optimal with 94% overall accuracy (134/142 total replicates). These conditions correctly classified strains in most instances, with 2 major errors and 6 very major errors (Table 2, Fig 2, S2 Fig).

In the case of cefepime, a 2 h incubation with 4 μg/mL antibiotic resulted in a 96% overall accuracy (128/134 total replicates), with 3 major errors and 3 very major errors (Table 2, Fig 3, S3 Fig). We excluded strains BAA-39 and HFH-29568 from the statistical analysis as previous classification characterized the strains as intermediate resistance by Etest (MIC = 24 μg/mL). As previously noted, the MBT-ASTRA assay cannot be used to classify intermediate resistance in its current configuration [17]. Supporting this assertion, two of the independent replicates of the assay performed with strain BAA-39 gave a susceptible result while the other two gave a resistant result (Fig 3).

### Vancomycin resistance detection

A 2 h incubation with 2 μg/mL vancomycin showed optimal classification of resistance with this antibiotic; however, availability of resistant isolates limited testing to two strains, both with MICs > 256 μg/mL. These optimized conditions yielded an overall accuracy of 96% (119/124 replicates), with 5 major errors and no very major errors (Table 2, Fig 4a, S4 Fig). As with cefepime, we observed ambiguous results with intermediate resistance phenotypes and therefore excluded these isolates from the analysis. The 4 strains present in the panel classified as displaying intermediate resistance by Etest categorized nearly uniformly as susceptible under these conditions (Fig 4a). Given the clinical importance of vancomycin-intermediate *Staphylococcus aureus* (VISA) isolates, we obtained an additional VISA-specific panel from the CDC and tested this panel under the same assay conditions. Testing of these 14 additional strains resulted in one called as susceptible for all four independent replicates (strain AR# 0222) while the remainders classified as resistant in three or four of the replicates (Fig 4b).

### Assay reproducibility

To better understand assay reproducibility within single strains, we performed an additional 40 replicates with at least two susceptible and two resistant strains for each antibiotic, as well as with intermediate resistance strains when available (Fig 5). Testing across all four antibiotics for fully susceptible or resistant strains showed an overall sensitivity of 98% and a specificity of 92%, with a corollary error rate of 5% (33 errors from 680 replicates, excluding intermediate resistance). However, the distribution of errors was not even across antibiotics; ciprofloxacin (9% error rate) and cefepime (8% error rate) showed the majority of errors while oxacillin yielded no errors (Fig 5). Interestingly, the vast majority of errors in this data set were major errors (28 of 33, 85% of all errors) with only 5 very major errors across all 680 replicates (0.7%). Unlike the assay validation reported in the previous three subsections, up to 12 replicates were performed from individual starter cultures. There appeared to be a loose correlation in relative growth values between replicates derived from an individual starter culture with multiple errors resulting from one starter culture and not another from a given set. We observed this mainly with susceptible strains and this appeared to be the result of higher than expected signal intensity from antibiotic treated samples.

Results from the larger number of replicates for strains with intermediate resistance were in line with the previous results and highlight the difficulties of applying the MBT-ASTRA assay in its current form to intermediate resistance. Strains BAA-39 with cefepime and HIP13170 with vancomycin both performed similarly to the fully susceptible strains for those respective antibiotics, despite the assay being optimized to employ the lowest antibiotic concentration possible while consistently classifying susceptible strains correctly. In contrast, performing the assay with vancomycin on strain AR# 0217 resulted in a widely scattered distribution of relative growth values centered on approximately 0.75 but ranging from nearly zero to greater than one (Fig 5).

### Performance with spiked blood culture

**Fig 6:**
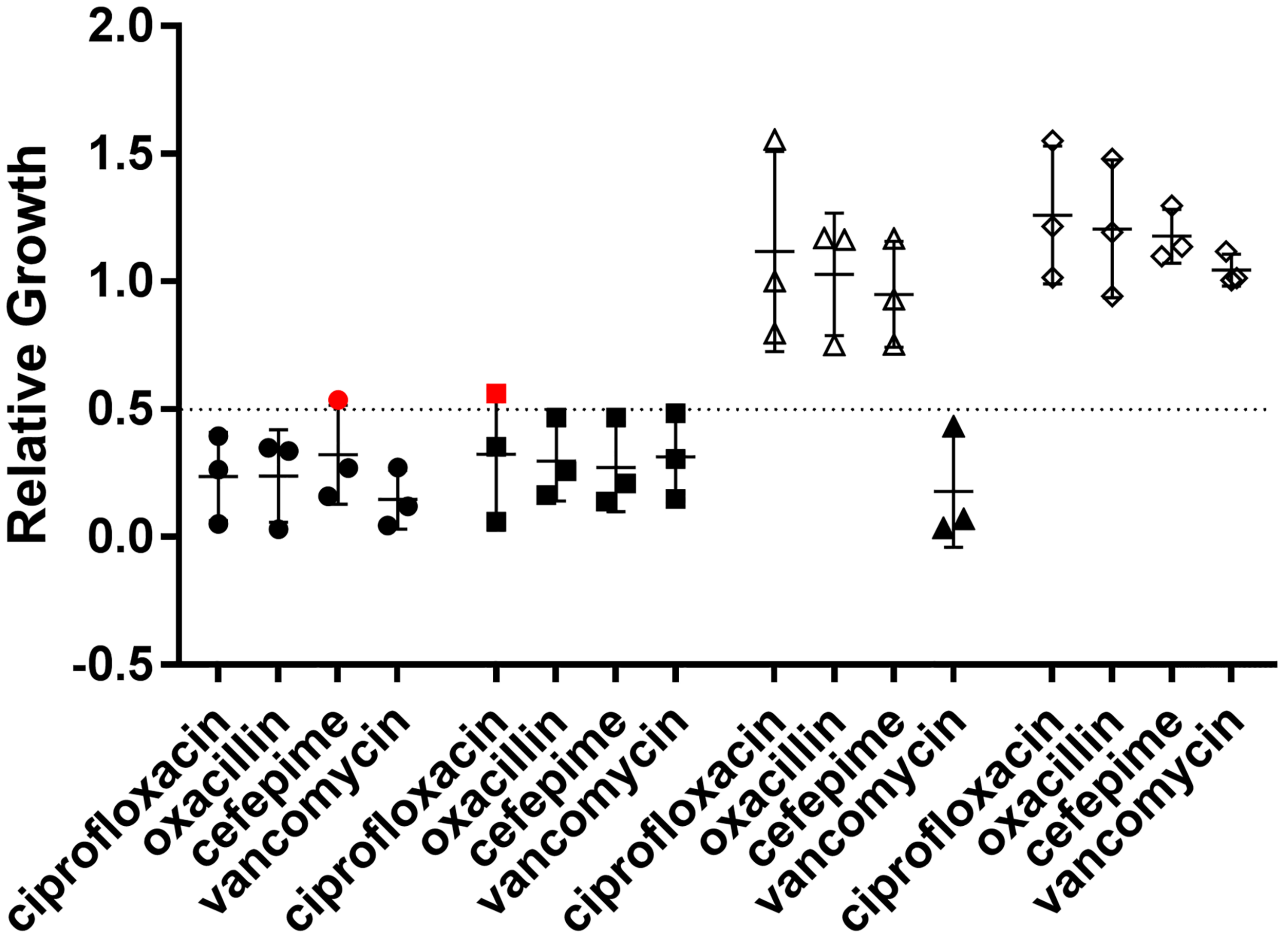
Assay performance on blood culture. MBT-ASTRA assay performed from spiked blood culture. Relative growth values from each replicate are shown, with data points representing major errors colored red. Strain FDAARGOS_159 represented by (●), strain ATCC 25923 represented by (■), strain BAA-44 represented by (▲), strain BR-VRSA represented by (♦). Filled symbols indicate the strain is susceptible to the antibiotic by reference standard while open symbols indicate resistance. A relative growth cutoff of 0.5 was utilized to classify resistance, indicated by the horizontal line. For each strain, 3 independent replicates were obtained on different days, with the mean and standard deviation shown for each set.

Finally, we assessed the performance of the MBT-ASTRA assay optimized for *S*. *aureus* with positive blood cultures. Four strains representing susceptible and resistant isolates were selected and spiked into blood culture bottles. After the blood cultures flagged positive, we removed a small portion of the cultures and utilized them for the MBT-ASTRA assay, with the inclusion of an additional washing step after the 2 h incubation to remove blood cells. Blood culture testing of three independent replicates performed for each strain with all four antibiotics yielded an accuracy of 96%, with two major errors and no very major errors (Fig 6). An averaging of the data points across strain and antibiotic combinations resulted in the correct classification in each instance. Despite the presence of additional peaks originating from the spiked-in standard, we observed correct species identification from every no-antibiotic sample via the Bruker Biotyper program with scores all above the cutoff of 2.0 for species assignment.

## Discussion

MALDI-TOF represents an attractive platform for the development of AST assays due to its rapid sample analysis speed and low operating costs. MALDI-TOF instruments are also already widely available in clinical microbiology laboratories, which would facilitate adoption of new diagnostic assays utilizing the platform. Semi-quantitative MALDI-TOF, in the form of the MBT-ASTRA assay, is a promising route forward; however, previous studies only applied the assay to Gram-negative and mycobacteria species. The results presented here demonstrate for the first time that the resistance profile of a Gram-positive species, *S*. *aureus*, can also be properly determined. We obtained an overall accuracy of approximately 95% across 35 strains for each antibiotic tested, which is marginally lower than the accuracies noted in previous studies for Gram-negatives and mycobacteria. We show here this technique is also applicable for AST directly from blood culture through mock clinical testing. In contrast to most previous reports describing the MBT-ASTRA assay [16, 19], this study documents the precision of this assay across multiple replicates for each strain and antibiotic combination. This allowed for in depth analysis of biological and technical variability and for the identification of possible causes of errors.

Gram-positive bacteria present a challenge for analysis by MALDI-TOF due to the thick peptidoglycan layer which has been noted to interfere in cell lysis in previous studies [5]. Here, similar issues arose. Specifically, manual examination of the MS spectra from replicates resulting in erroneous classification indicated that a portion of these failures occurred during the protein extraction step, likely due to a lack of proper cell lysis. This was further evidenced by the presence of a visibly large cell mass prior to the extraction step that putatively induced the observed poor signal intensity during the MALDI analysis. The possibility that failure occurred during sample spotting, matrix deposition, or spectral acquisition is unlikely, as positive control spike-ins for RNase A after protein extraction showed peaks with intensities in the normal range.

Previous studies on the MBT-ASTRA assay employed a minimum AUC cutoff for samples without antibiotic, which would potentially obviate the problem of extraction failure for susceptible strains. In optimizing the assay for Gram-positives, we chose to omit an AUC cutoff as *S*. *aureus* in general produced relatively low AUC values. While useful in certain aspects, implementing an AUC cutoff here would have filtered out data for the slower growing strains, especially the VISA isolates, which consistently resulted in very low signal intensities. Additionally, an AUC cutoff would be ineffective for preventing very major errors (reference result is resistance and assay result is susceptible) resulting from an extraction failure on the antibiotic treated sample from a resistant strain.

Extraction failures that led to errors in resistance classification appeared to increase in frequency with increased cell mass, although the correlation was not perfect with other factors likely also contributory. This was noted during the optimization stage for each antibiotic when incubation times beyond 2 h were evaluated and was more notable for faster growing strains. In addition to directly causing assay failure, this issue placed a limit on incubation time, thereby restricting further assay optimization to improve overall performance for slow growing isolates. In this context, future implementation of methods to improve extraction efficiency for the MBT-ASTRA assay would mitigate this issue. Our future optimization of this technique will focus on the addition of enzymatic pretreatment, thermal lysis, or physical disruption steps, or a combination thereof. Exploratory experiments with physical disruption via glass beads in our laboratory have so far shown some success in this regard.

Beyond extraction failure, another source of error appeared to stem from variability of independent cultures of the same strain displaying slightly more or less sensitivity to an antibiotic based on growth rate quantification by MALDI-TOF. Analysis of multiple replicates with and without antibiotics generated from a single starter culture highlighted this phenomenon. In these cases, the relative growth values derived from these replicates would trend similarly in a certain direction. The replicates of FDAARGOS_159 treated with ciprofloxacin performed to test assay reproducibility provide an example of this grouping: the mean of twelve replicates from a single starter culture was significantly higher than the mean of the rest of the replicates generated from other starter cultures (p = 0.023, see S5 Fig for more details). These differences in apparent antibiotic sensitivity could originate from several factors, including simple variability in technician performance. However, different sets of replicates generated by the same technician at the same time did not display obvious trends in relative growth groupings. Another possible cause could be subtle differences in growth or sample preparation conditions, such as variances between batches of media or formic acid preparations. Finally, while unlikely, it is possible individual colonies used for starting cultures developed minor mutations that effected antibiotic sensitivity. Regardless of the cause, this issue could potentially decrease the utility of performing multiple replicates of the assay as a method to mitigate single incorrect classification. This is true unless multiple, different specimens are available (e.g., blood culture sampling from different sites) that could provide confirmatory diagnoses from diverse sites as opposed to technical replicate confirmation.

In this study, we did not include the results from strains displaying intermediate resistance for a given antibiotic in the accuracy or error analysis, as the MBT-ASTRA assay is not currently designed to classify these strains. Modifications to the assay that would allow classification of intermediate resistance have been discussed elsewhere and include utilizing multiple antibiotic concentrations or finding a correlation between relative growth and MIC [17]. While the former may be feasible, correlations between relative growth and MIC were not always observed in previous cases [16, 18] or in this work. Correct classification of VISA isolates may be especially challenging, given the variety of mutations leading to intermediate vancomycin resistance and the various degrees of growth rate depression imparted by them [24].

Improvements to the assay for *S*. *aureus* are likely feasible even without major modifications. The most valuable change would be the addition of a more effective protein extraction step followed by further optimization, potentially including the use of higher antibiotic concentrations coupled with longer incubation times to allow more distinct differences in growth for susceptible strains. In this context, an extended incubation time may likewise make the introduction of an AUC cutoff appropriate, resulting in some anomalous results being discarded. The choice of 0.5 for the relative growth cutoff was also chosen empirically and could be further optimized; alternatively, the analysis could be altered to classify samples with a relative growth between certain values (e.g., between 0.4 and 0.6) as ambiguous to avoid erroneous calls.

In conclusion, we demonstrate the first use of the MBT-ASTRA assay for AST in a Gram-positive bacterium. Despite the difficulties discussed above, the assay performed adequately in *S*. *aureus* for clinical application with both standard culture and blood culture, with an overall accuracy rate of 95%. Further studies in additional organisms and antibiotics are warranted to extend the utility of this assay to a wider range of Gram-positive bacteria.

## Supporting information

S1 Fig The ciprofloxacin MICs of each strain are plotted against the average of the relative growth values determined through the MBT-ASTRA assay. A relative growth cutoff of 0.5 was utilized to classify resistance, indicated by the horizontal line. Vertical lines are drawn at the susceptibility and resistance breakpoints (susceptible ≤ 1 μg/mL; resistant ≥ 4 μg/mL).
(TIF)

S2 Fig The oxacillin MICs of each strain are plotted against the average of the relative growth values determined through the MBT-ASTRA assay. A relative growth cutoff of 0.5 was utilized to classify resistance, indicated by the horizontal line. A vertical line is drawn at the susceptibility breakpoint (susceptible ≤ 2 μg/mL).
(TIF)

S3 Fig The cefepime MICs of each strain are plotted against the average of the relative growth values determined through the MBT-ASTRA assay. A relative growth cutoff of 0.5 was utilized to classify resistance, indicated by the horizontal line. Vertical lines are drawn at the susceptibility and resistance breakpoints (susceptible ≤ 8 μg/mL; resistant ≥ 32 μg/mL). Strains falling between these values are classified as intermediate resistance.
(TIF)

S4 Fig The vancomycin MICs of each strain are plotted against the average of the relative growth values determined through the MBT-ASTRA assay. A relative growth cutoff of 0.5 was utilized to classify resistance, indicated by the horizontal line. Vertical lines are drawn at the susceptibility and resistance breakpoints (susceptible ≤ 2 μg/mL; resistant ≥ 16 μg/mL). Strains falling between these values are classified as intermediate resistance.
(TIF)

S5 Fig The replicates of FDAARGOS_159 treated with ciprofloxacin performed to test assay reproducibility (Fig 5) are shown. Group 1 represents a set of 12 replicates generated from a single starter culture while group 2 represents the remaining replicates. Of the 12 replicates in group 1, 10 showed a relative growth above 0.4 (5 of which were incorrectly classified). Meanwhile, only 6 of the remaining 28 replicates resulted in relative growth values above 0.4. The relative growth mean of group 1 (0.428) was significantly different than that of group 2 (0.291; p = 0.023, from unpaired t test with Welch’s correction).
(TIF)
