# Supplementary figures and images for: Semi-quantitative MALDI-TOF for antimicrobial susceptibility testing in Staphylococcus aureus
Source: PLoS One. 2017 Aug 31;12(8):e0183899. doi: 10.1371/journal.pone.0183899 (PMC5578647; doi:10.1371/journal.pone.0183899)

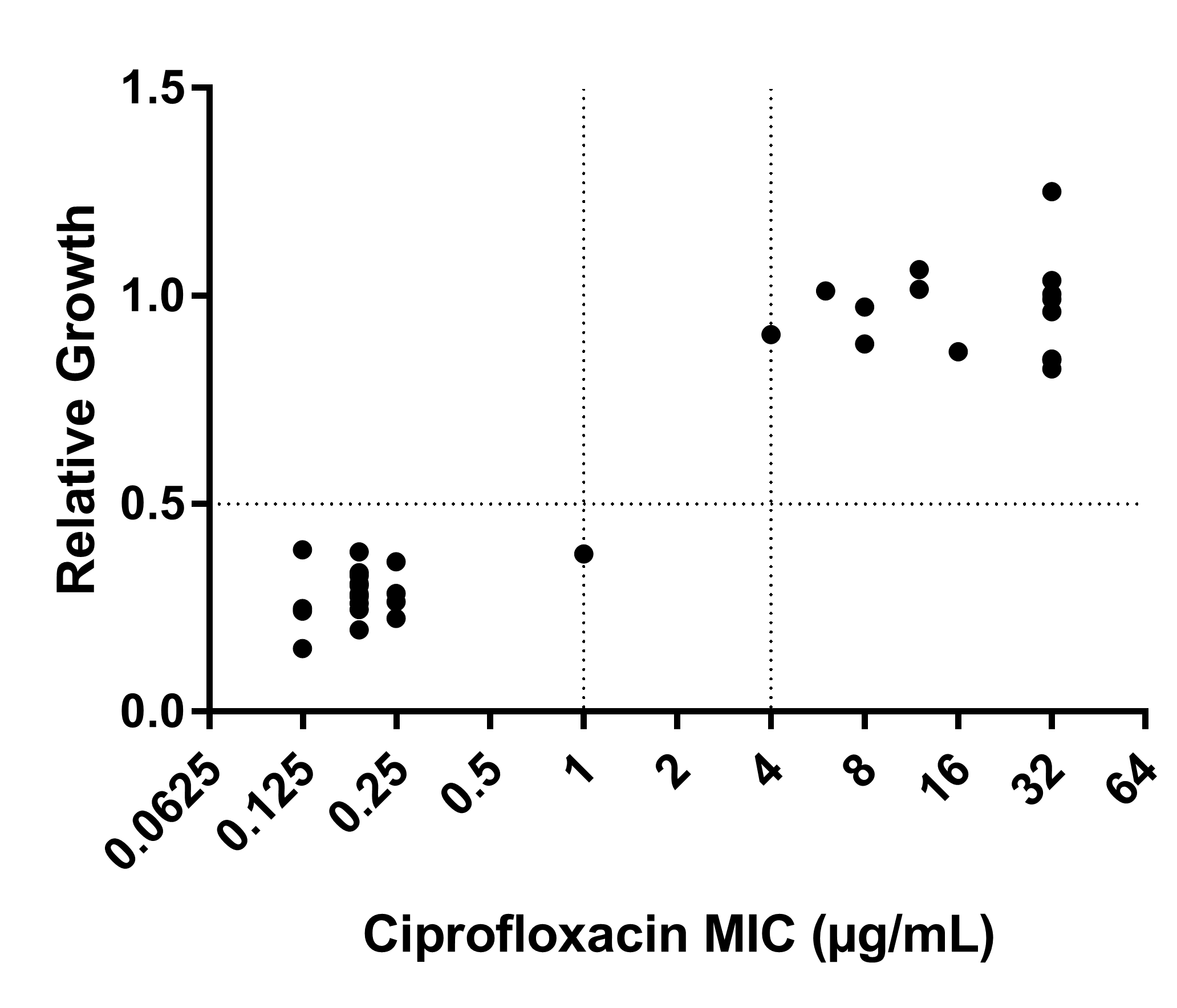

Supplement: S1 Fig — A relative growth cutoff of 0.5 was utilized to classify resistance, indicated by the horizontal line. Vertical lines are drawn at the susceptibility and resistance breakpoints (susceptible ≤ 1 μg/mL; resistant ≥ 4 μg/mL). (TIF) [file pone.0183899.s001.tif]

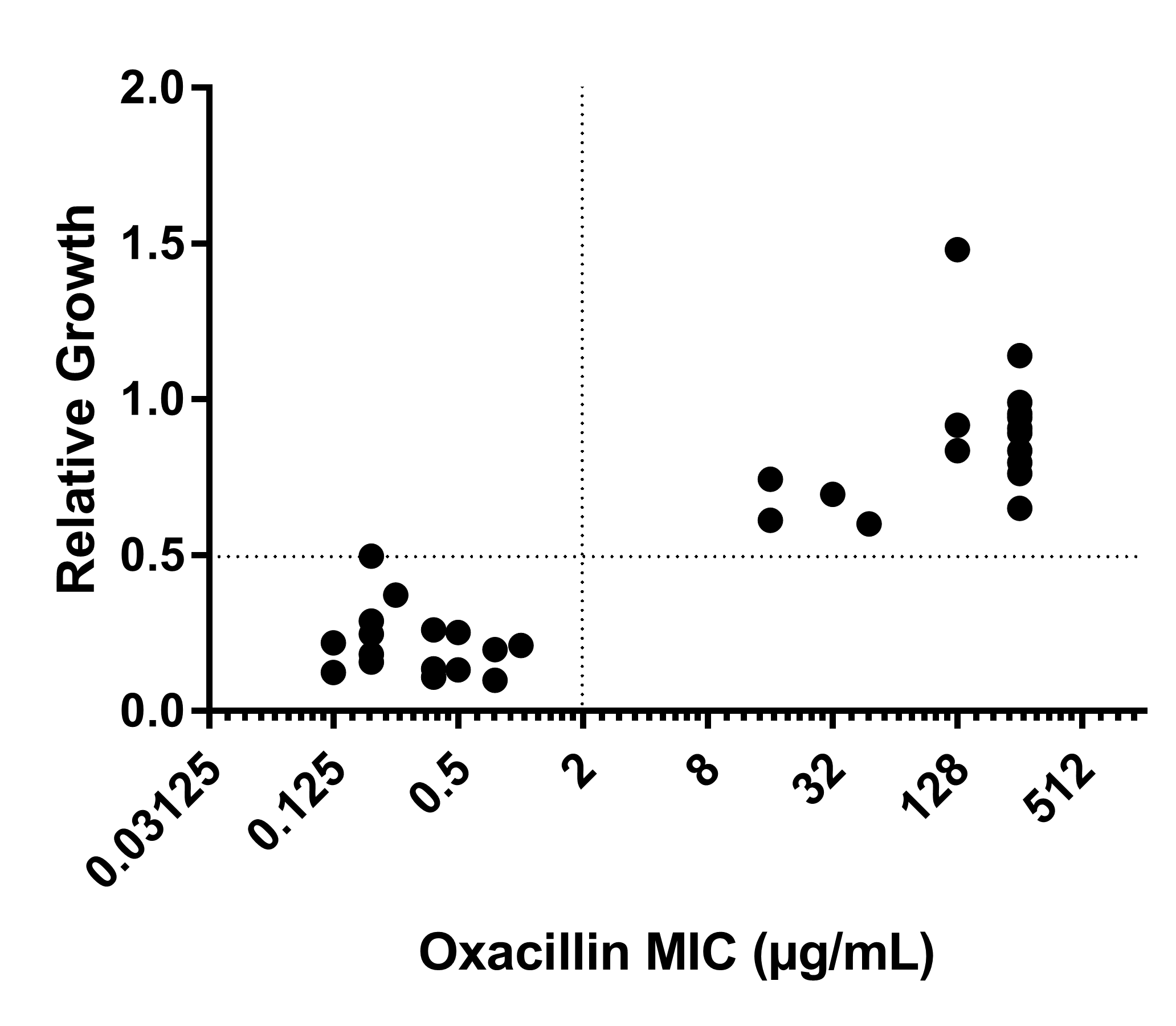

Supplement: S2 Fig — A relative growth cutoff of 0.5 was utilized to classify resistance, indicated by the horizontal line. A vertical line is drawn at the susceptibility breakpoint (susceptible ≤ 2 μg/mL). (TIF) [file pone.0183899.s002.tif]

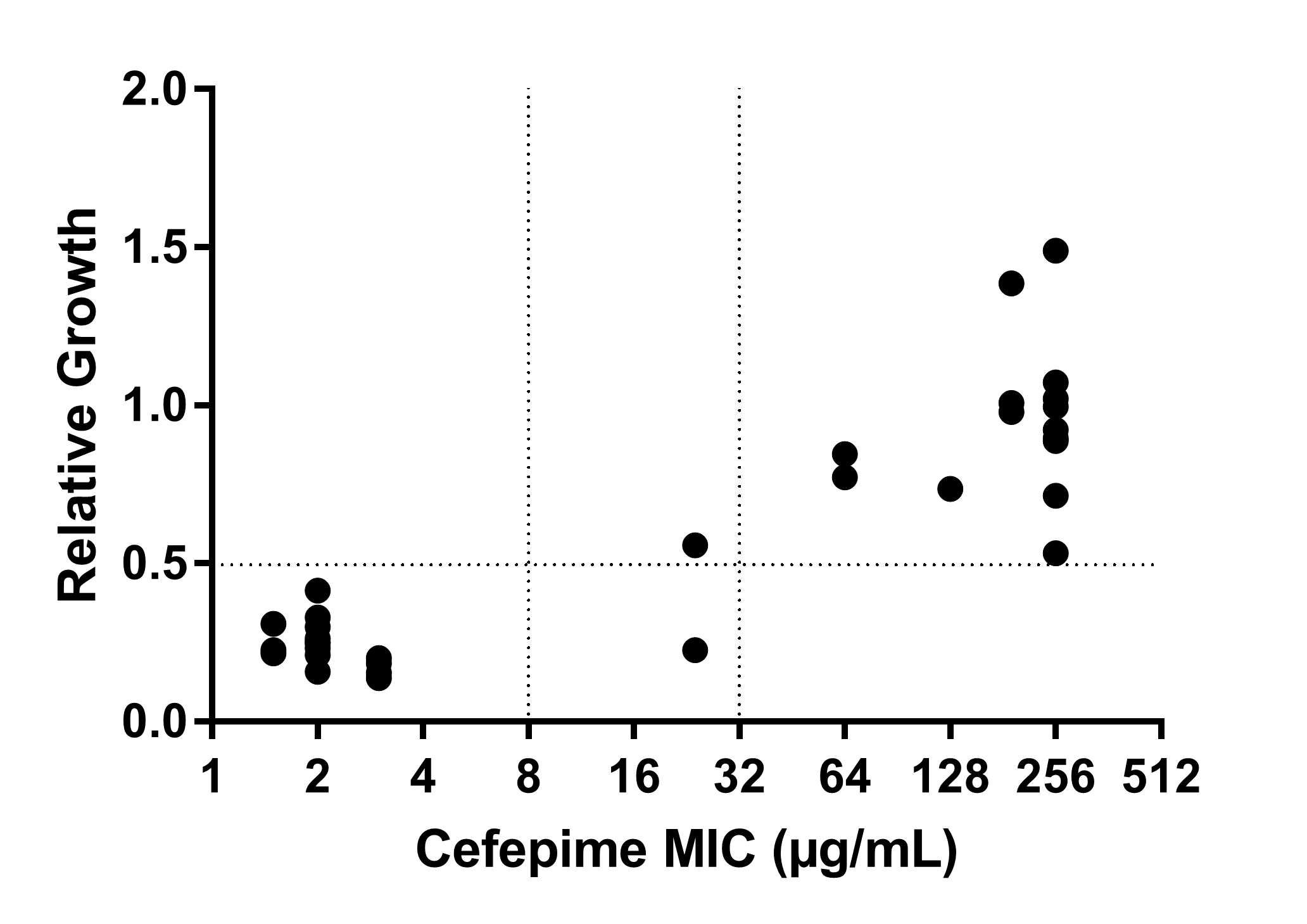

Supplement: S3 Fig — A relative growth cutoff of 0.5 was utilized to classify resistance, indicated by the horizontal line. Vertical lines are drawn at the susceptibility and resistance breakpoints (susceptible ≤ 8 μg/mL; resistant ≥ 32 μg/mL). Strains falling between these values are classified as intermediate resistance. (TIF) [file pone.0183899.s003.tif]

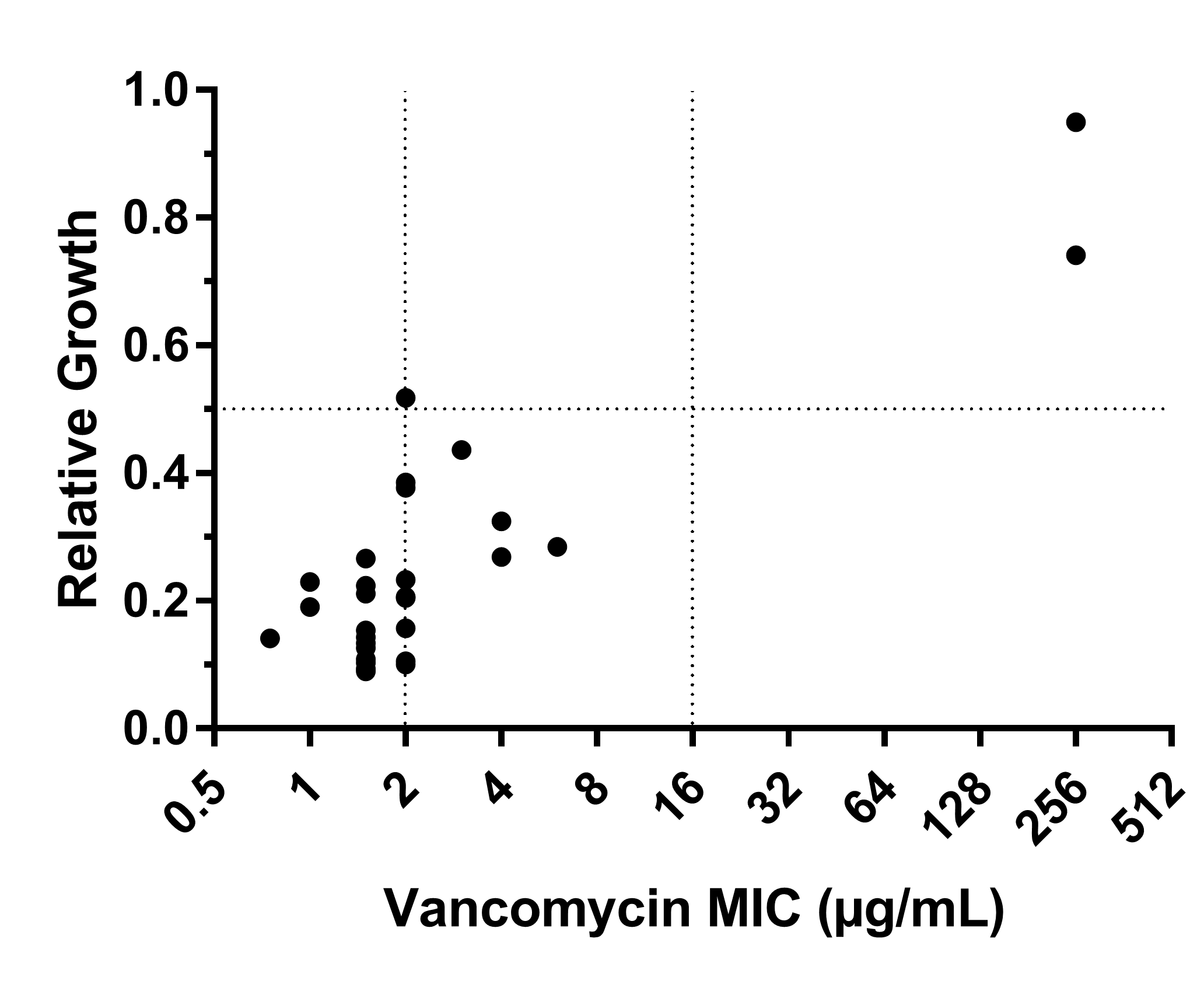

Supplement: S4 Fig — A relative growth cutoff of 0.5 was utilized to classify resistance, indicated by the horizontal line. Vertical lines are drawn at the susceptibility and resistance breakpoints (susceptible ≤ 2 μg/mL; resistant ≥ 16 μg/mL). Strains falling between these values are classified as intermediate resistance. (TIF) [file pone.0183899.s004.tif]

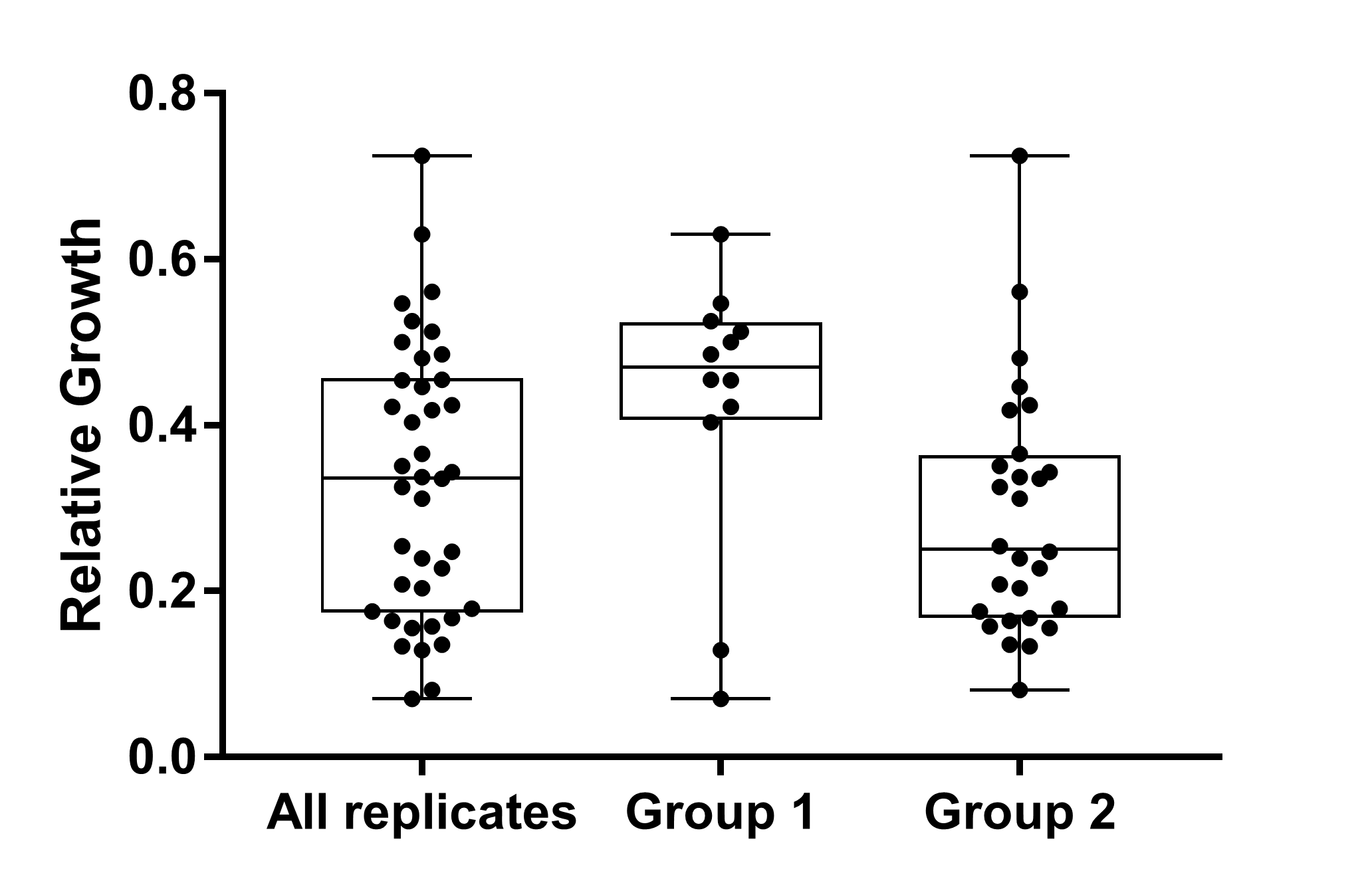

Supplement: S5 Fig — Group 1 represents a set of 12 replicates generated from a single starter culture while group 2 represents the remaining replicates. Of the 12 replicates in group 1, 10 showed a relative growth above 0.4 (5 of which were incorrectly classified). Meanwhile, only 6 of the remaining 28 replicates resulted in relative growth values above 0.4. The relative growth mean of group 1 (0.428) was significantly different than that of group 2 (0.291; p = 0.023, from unpaired t test with Welch’s correction). (TIF) [file pone.0183899.s005.tif]
